# The Relationship Between Fat-Free Mass and Glucose Metabolism in Children and Adolescents: A Systematic Review and Meta-Analysis

**DOI:** 10.3389/fped.2022.864904

**Published:** 2022-04-26

**Authors:** Lijun Wu, Fangfang Chen, Junting Liu, Dongqing Hou, Tao Li, Yiren Chen, Zijun Liao

**Affiliations:** ^1^Department of Epidemiology, Capital Institute of Pediatrics, Beijing, China; ^2^Child Health Big Data Research Center, Capital Institute of Pediatrics, Beijing, China; ^3^Department of Integrated Early Childhood Development, Capital Institute of Pediatrics, Beijing, China

**Keywords:** fat-free mass, children, glucose metabolism, systematic review, meta-analysis

## Abstract

**Purpose:**

To assess the relationship between fat-free mass (FFM) and glucose metabolism in children 0–18 years of age.

**Methods:**

We performed a systematic review of the literature on Medline/PubMed, SinoMed, Embase, and the Cochrane Library using the PRISMA 2020 guidelines to 12 October 2021; this encompassed observational studies in which the relationship between FFM and glucose metabolism was assessed. Correlation coefficient (*r*), regression coefficient (β), and odds ratio (OR) values in the studies were extracted and recorded as the primary data. “Agency for Healthcare Research and Quality” quality-assessment forms recommended for cross-sectional/prevalence studies were applied to evaluate the quality of the selected studies, and we executed R software to combine the pooled data.

**Results:**

We included eight studies comprising 13,282 individuals, five of which involved the assessment of the relationship between FFM and blood glucose, and four on the relationship between FFM and insulin resistance (IR). Our results showed that FFM was significantly associated with fasting plasma insulin levels (*r* = 0.34, 95% CI: 0.30–0.39, *P* < 0.001). Due to high heterogeneity or insufficient quantity of data, the studies of the relationship between FFM and fasting plasma glucose, HOMA-IR, or HbA1c were not congruent, and were therefore not suitable for meta-analysis.

**Conclusion:**

Our results indicated that FFM was significantly associated with fasting plasma insulin levels. As far as we have determined, this is the first-ever systematic review and meta-analysis of the associations between FFM and glucose metabolism in children and adolescents; and our results thus provide novel information to fill a gap in the literature in this area.

**Systematic Review Registration:**

https://www.crd.york.ac.uk/prospero/display_record.php?ID=CRD42020150320, PROSPERO CRD42020150320.

## Introduction

Type 2 diabetes (T2D) is one of the most common metabolic disorders. In 2014, 9% of adults exhibited the disease worldwide (1), and its prevalence continues to climb (2). Childhood obesity increases the risk of developing T2D (3), and the early prevention of childhood obesity may therefore be critical to T2D prevention (4).

It is well-recognized that skeletal muscle is an important component of body composition and a metabolic sink for glucose disposal (5). Skeletal muscle also plays an important role in the regulation of blood glucose levels and affects the development of insulin resistance (IR) and T2D (6). A previous study reported that decreased skeletal muscle mass is associated with deterioration of insulin sensitivity (7). A longitudinal study showed a positive association between abdominal muscle quantity and T2D (8), and a cross-sectional study revealed that trunk muscle quality was related to glucose tolerance (9). Recently, some studies have found similar results in the pediatric population (10, 11). Thus, targeting the mechanism(s) underlying skeletal muscle activity in glucose metabolism may prevent or delay IR and T2D.

There are several potential pathways that subserve the effects of skeletal muscle on glucose metabolism. (a) Skeletal muscle stores glucose as glycogen through the translocation of glucose transporter type-4 via insulin signals (12). (b) Testosterone may be critical to skeletal muscle glucose metabolism, as it has been reported that elevated muscular 5α-dihydrotestosterone may contribute to the improvement of hyperglycemia in T2D rats (13), and higher testosterone concentrations and muscle mass were associated with improved glucose metabolism in children (11). (c) Forkhead box class O transcription factors regulate glucose metabolism and markers of inflammation in skeletal muscle (14). Although skeletal muscle mass is negatively correlated with IR and T2D, a precise role for skeletal muscle mass in glucose metabolism has not yet been fully elucidated.

Because it is difficult to directly quantify the amount of muscle mass, body composition is usually divided into fat mass and fat-free mass (FFM) for evaluation. To the best of our knowledge, there is no extant meta-analysis on the relationship between FFM and glucose metabolism (blood glucose and IR) in children aged 0–18 years. Our study will therefore assist in clarifying the association between muscle mass and glucose metabolism in children and adolescents.

## Methods

The protocol we used for the present systematic review was based on PRISMA 2020 (Preferred Reporting Items for Systematic reviews and Meta-Analyses) (15) and was registered in PROSPERO (the International Prospective Register of Systematic Reviews; http://www.crd.york.ac.uk/prospero) under the registration number CRD42020150320.

We conducted our literature search using MEDLINE/PubMed, EMBASE, SinoMed, and the Cochrane Library; and restricted the search to (a) studies published in English or Chinese, and (b) studies on humans, with only samples from children and adolescents under 18 years of age.

Observational studies (of a cross-sectional or longitudinal design) that involved the relation between muscle mass/FFM and glucose metabolism in children and adolescents were then selected. To be included, studies were required to meet the following criteria: (a) muscle mass/FFM measured by a direct method, bioelectrical impedance analysis (BIA)/dual energy X-ray absorptiometry (DXA)/magnetic resonance imaging (MRI)/air-replaced method, or other method; and (b) assessment of at least one glucose metabolism indicator (fasting glucose, IR, homeostasis model assessment [HOMA-IR], or glycosylated hemoglobin A1c [HbA1c]).

Studies were not included if: (a) the study outcomes only focused on surrogate indicators of body composition or physical fitness rather than directly measured indicators; (b) they assessed the relationship in adults or non-human species; (c) they involved children with severe physical limitations; (d) they referred to children with disorders that could limit generalizability (e.g., those manifesting mitochondrial dysfunction; Prader-Willi syndrome; non-alcoholic fatty-liver disease; polycystic ovarian syndrome; or mental disorders that included attention-deficit/hyperactivity disorder [ADHD]; conduct or neuropsychiatric disorders that included schizophrenia; or any detected delay in communication, adaptive cognition, or socio-emotional domains); and (e) if they referred to a particular population group such as an aboriginal group, immigrant group; or to a specific economic status or to an unhealthy child.

### Data Extraction (Selection and Coding)

#### Risk of Bias (Quality) Assessment

We used “Agency for Healthcare Research and Quality (AHRQ, USA)” quality-assessment forms containing 11-item checklists that are recommended for cross-sectional/prevalence studies to assess the methodological quality of the selected studies, and used two independent researchers (Fangfang Chen and Junting Liu). Any disagreements over the assessment of bias risk were discussed, and a consensus was reached, with a third researcher (Dongqing Hou) consulted if consensus could not be attained.

An item was scored “0” if it was answered “NO” or “UNCLEAR,” and “1” if it was answered “YES.” Article quality was assessed as follows: low quality, 0–3; moderate quality, 4–7; and high quality, 8–11.

### Strategy for Data Synthesis

The amount of observed variance that reflected actual differences in effect size across the included trials was graded using the *I*^2^ statistic, with values representing mild, moderate, and severe heterogeneity (<25, 25–75, and > 75%, respectively) (16). We clustered subgroup analysis by sex and processed these data according to the results of the heterogeneity test and study quality. The correlation coefficients (*r*) and regression coefficients (β) for FFM and glucose metabolism (fasting plasma glucose [FPG], fasting plasma insulin [INS] and HOMA-IR [= FPG*FPI/22.5]) were then synthesized, and Tables were created to summarize the characteristics of the selected studies. The feasibility of conducting a meta-analysis was thus determined after the data were extracted.

R software was used to combine the pooled data. A fixed-effect model was exploited when there was no evidence of heterogeneity; otherwise, a random-effect model was applied.

### Analysis of Subgroups or Subsets

Subgroup and meta-regression analyses were carried out on the main factors causing heterogeneity, including the sex of the study participants. Furthermore, the design and methodological quality of the studies were considered for additional subgroup analyses.

## Results

### Identification and Selection of Studies

**Figure 1:**
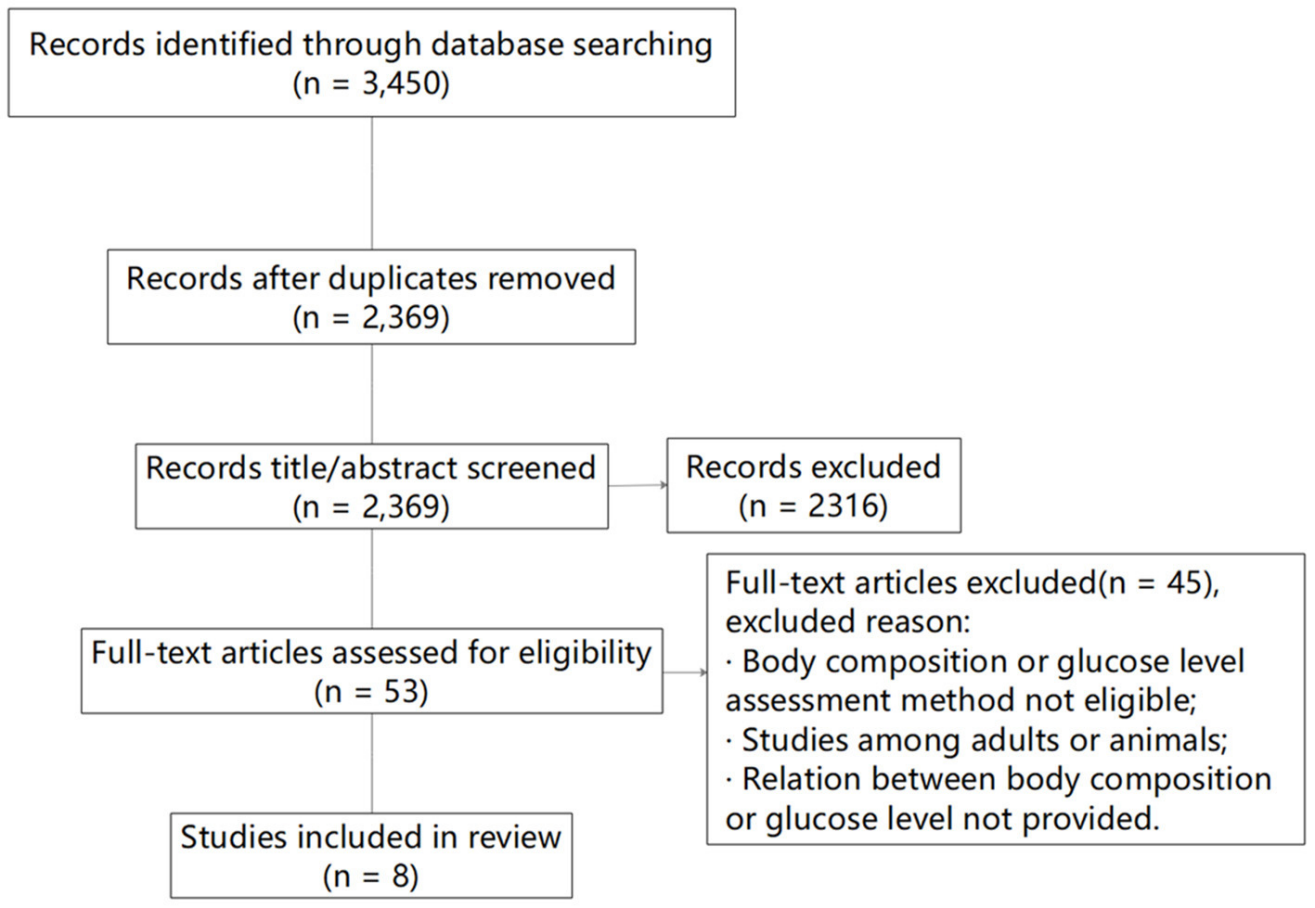
Flow diagram of the literature search and paper selection process.

The original search identified 3,450 studies, and after removal of duplicates and the elimination of papers based upon eligibility criteria, eight studies remained (Figure 1). Of the eight studies, the relationship between FFM and FPG was assessed in five, four involved an analysis between FFM and INS, four studies focused on the relationship between FFM and HOMA-IR, and in one study a linear analysis (β) was performed between FFM and HbA1c.

### Study Characteristics

The characteristics of all eight studies are shown in Table 1. Three papers based on two studies (baseline and follow-up data were used separately) were conducted in the UK (study ID: 3, 4, 6), two in the U.S. (study ID: 2, 7), two in China (study ID: 1, 5), and one study in Brazil (study ID: 8). Table 2 depicts the quality evaluation of the methodology according to AHRQ quality-assessment forms (US 2004). In the case of AHRQ scoring, there were 2 studies with 8 points, 1 study with 7 points, 2 studies with 6 points, and 3 studies with 5 points. There were 2 high quality trials with 8 points and 6 moderate quality trials with an average score of 5.7 points.

**Table 1:** Characteristics of the included trials.

**Study ID**	**References**	**Study year**	**Study location(s)**	**Study design**	**Sample size (total, M/F)**	**Age (years)**	**Body composition assessment**	**Which bio-marker related with FFM were reported**	**Indices of correlation**
1	(17)	2013–2015	Beijing, China	Cross sectional study	7926, 4036/3890	6–17	DXA	FPG	r, β
2	(18)	Unclear	Philadelphia, USA	Cross-sectional	36, 15/21	6–18	DXA	FPG, INS, HOMA	r
3	(19)	2000–2001	Plymouth, UK	Cohort study, baseline data was used	234, 133/101	5.9 ± 0.3	BIA	FPG, HOMA	r
4	(4)	2004–2007	England	Cross-sectional study	4633, 2237/2396	9–10	BIA	FPG, HOMA, HbA1c	β
5	(20)	2010	Hongkong, China	Cross-sectional study	40,20/20	12.9 ± 0.1	DXA	FPG, INS	β
6	(21)	2000–2001	Plymouth, UK	Cohort study	272, 152/120	7–12	DXA	INS	r
7	(22)	Unclear	USA	Cross-sectional study	92, 33/59	13–17	DXA	INS	r
8	(23)	2004	São Paulo, Brazil	Cross-sectional study	49, 12/37	16.6 ± 1.4	DXA	HOMA	β

*M, male; F, female; DXA, dual energy X-ray absorptiometry; BIA, bioelectrical impedance analysis; FFM, fat free mass; FPG, fasting plasma glucose; INS, insulin; HOMA, homeostasis model assessment; HbA1c, glycosylated hemoglobin A1c*.

**Table 2:** The quality evaluation of the methodology according to Agency for Healthcare Research and Quality (AHRQ, US 2004) assessment forms*.

**Study ID**	**References**		**AHRQ 11-item checklist**											**Total score**	**Total score**
			**A**	**B**	**C**	**D ^#^**	**E**	**F**	**G**	**H**	**I**	**J**	**K**		
1	(17)		1	1	1	1	0	1	1	1	0	1	0	8	High quality
2	(18)		1	1	0	1	0	1	0	1	0	0	0	5	Moderate quality
3	(19)		1	1	1	1	0	1	0	1	0	1	1	8	High quality
4	(4)		1	1	1	1	0	1	0	1	0	1	0	7	Moderate quality
5	(20)		1	1	1	1	0	1	0	1	0	0	0	6	Moderate quality
6	(21)		1	0	0	1	0	0	0	1	0	1	1	5	Moderate quality
7	(22)		1	1	0	1	0	1	1	1	0	0	0	6	Moderate quality
8	(23)		1	1	0	1	0	0	1	1	0	0	0	5	Moderate quality

*1 = yes; 0 = no or unclear.

# *Study 1–8 were all population-based*.

*A, Define information source; B, List inclusion/exclusion criteria; C, Indicate identifying patients time; D, Indicate subjects consecutive or population-based; E, subjective components masked; F, Quality assessments; G, Patients exclusions explained; H, Confounding assessed and/or controlled; I, Handle missing data; J, Summarize data completeness; K, Clarify follow-up*.

*Article quality was assessed according to the total score as follows: low quality = 0–3; moderate quality = 4–7; high quality = 8–11*.

### Relationship Between FFM and FPG

**Figure 2:**
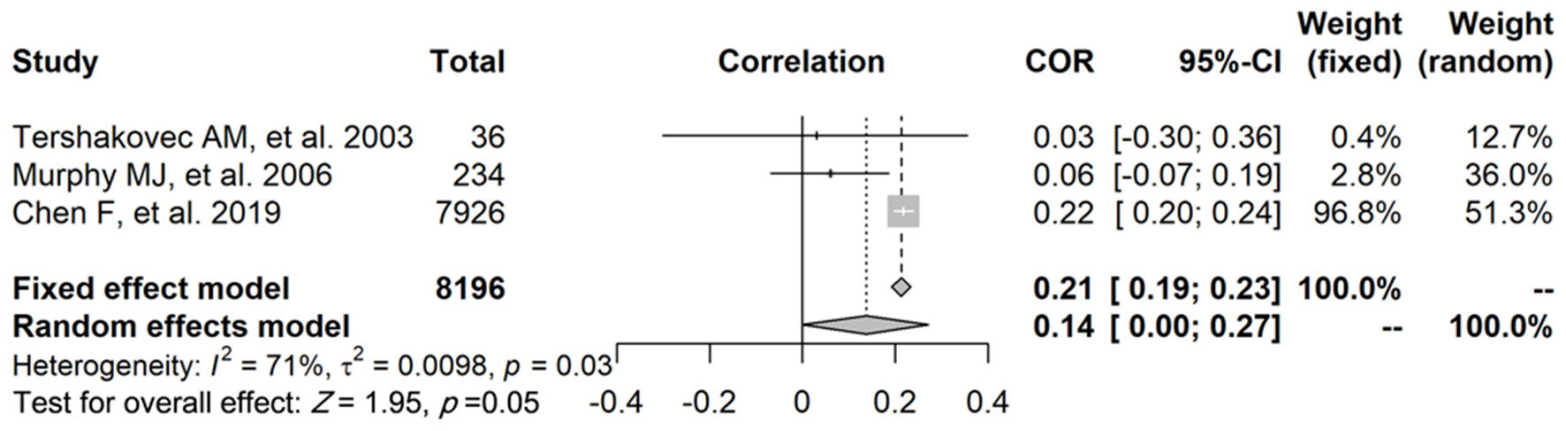
The relationship between FFM and FPG, and three studies provided the correlation coefficient (r).

**Figure 3:**
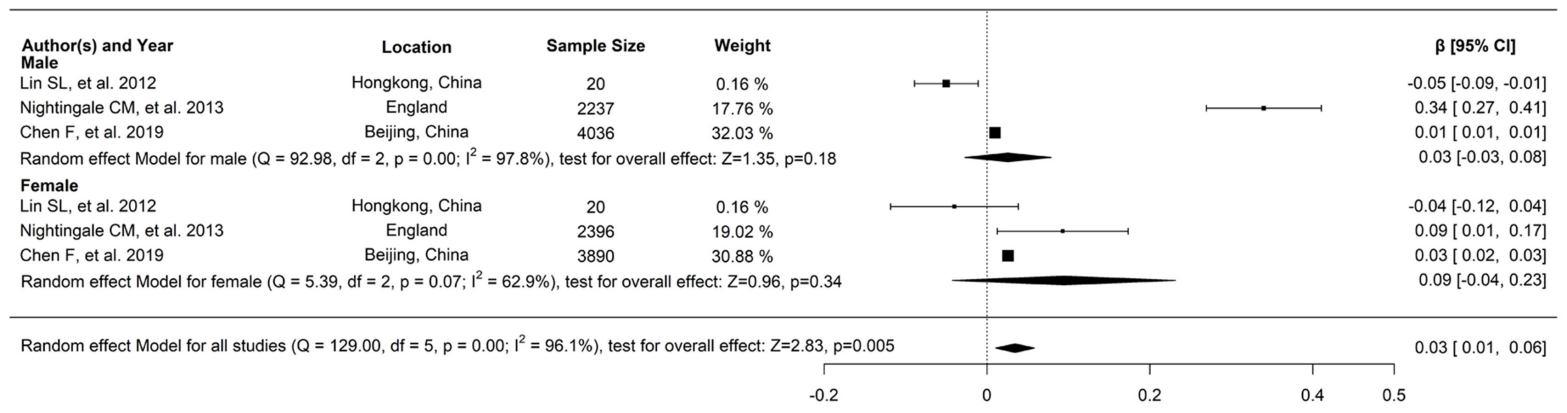
The relationship between FFM and FPG, and three studies provided the regression coefficient (β) through linear analysis.

Regarding the relationship between FFM and FPG, three studies reported a correlation coefficient (*r*) (study ID: 1, 2, 3) (Figure 2): there was no statistically significant association found between FFM and FPG in two of these studies (study ID: 2, 3), and Chen et al. (17) (study ID: 1) reported inconsistent results. Three studies also furnished a regression coefficient (β) through linear analysis (study ID: 1, 4, 5) (Figure 3). While Lin et al. (20) (study ID: 5) reported that FFM was negatively associated with FPG, studies by Chen et al. (17) (study ID: 1) and Nightingale et al. (4) (study ID: 4) revealed that FFM was positively associated with FPG. Because of high heterogeneity (*I*^2^ = 71 and 96.1%, respectively), we deemed these studies as not suitable for meta-analysis.

### Relationship Between FFM and INS

**Figure 4:**
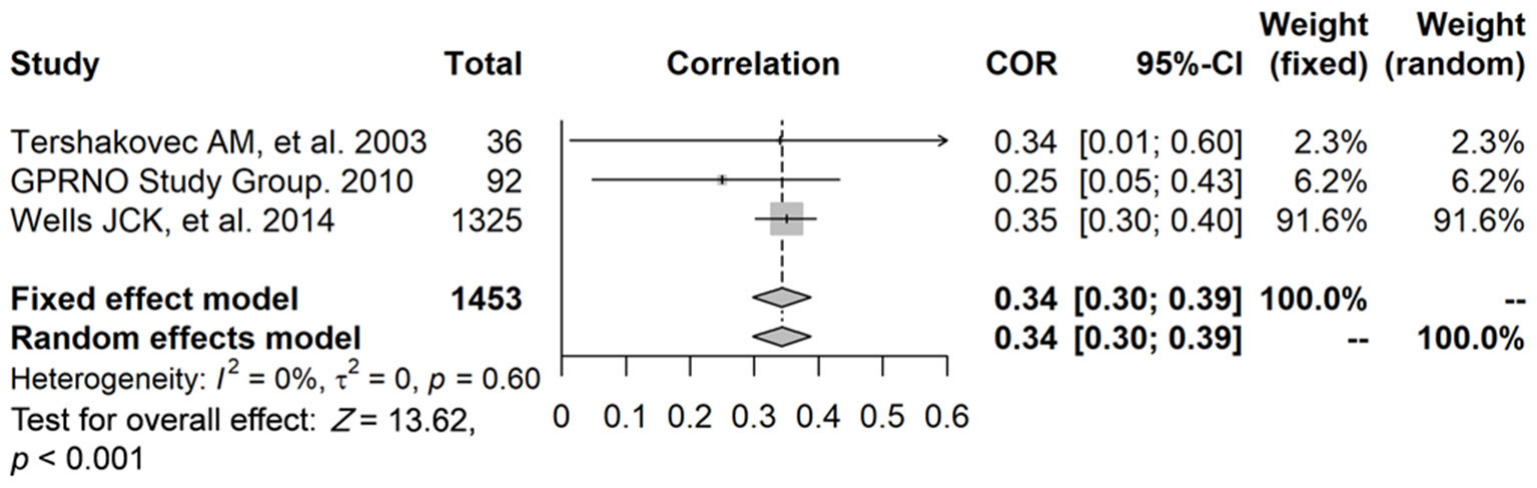
The relationship between FFM and fasting plasma insulin levels.

The data of three studies (*n* = 1,453) were pooled, and the results showed that, after data pooling using a fixed-effect model, FFM was significantly associated with fasting plasma insulin levels (Figure 4) (*r* = 0.34, 95% confidence interval [CI]: 0.30–0.39, *P* < 0.001).

In addition, Tershakovec et al. (18) (study ID: 2) reported that FFM was significantly associated with HOMA-IR (*r* = 0.32, *P* = 0.08), while, in contrast, Murphy et al. (19) (study ID: 3) reported the negative results (*r* = −0.15, *P* > 0.05, in males; *r* = 0.02, *P* > 0.05, in females). Santos et al. (23) (study ID: 8) showed that the final linear model regression coefficients for FFM were associated with IR among post-pubertal obese adolescents (β = 37.01, *P* = 0.007), and Nightingale et al. (4) (study ID: 4) showed the associations between FFM and HOMA-IR (β = 35.6, *P* < 0.001, in males; β = 28.6, *P* < 0.001, in females), and the associations between FFM and HbA1c (β = *3.68, P* < 0.0001, in males; β = −0.51, *P* < 0.0001, in females). Because of insufficient quantity, these studies are not suitable for ourmeta-analysis.

## Discussion

Considering that skeletal muscle plays a major role in glucose metabolism, most previous research concerns the negative relationship of muscle mass with T2D (24–26). However, a recent study has shown that sarcopenia is a new complication of T2D (27), and there is a reverse causality between muscle mass and T2D in middle-aged and elderly individuals. In order to exclude the influence of confounding factors such as age and degenerative disease, the study of the pediatric population could be better to clarify the relationship between muscle mass and glucose metabolism. Our research is the first meta-analysis of the relationship between muscle mass and glucose metabolism in children and adolescents. The results indicated that FFM was positively associated with fasting plasma insulin levels in children and adolescents.

According to reference 17 (study ID: 1) and after stratifying for overweight and obesity, we evaluated the relationship between FFM and INS and found that FFM exerted a protective effect on INS in children with normal weight, but that among children who are overweight or obese, body composition was related to elevated blood pressure and dyslipidemia but not to INS. This suggests that the effect of FFM on INS is inconsistent among children of different weight statuses, and it is necessary to further verify and explore the mechanisms.

Childhood obesity increases the risk of abnormal glucose metabolism, and FFM plays an important role in glucose metabolism. Anthropometric measures such as body mass index (BMI) are widely used to evaluate adiposity owing to their feasibility and low cost, but its use is limited because it does not discriminate for FFM and fat mass (28). A higher BMI may arise not only from greater body fat, but also from higher lean mass and/or bone mass, making it an imperfect measure of adiposity (29, 30). Therefore, it is crucial to study the relationships between body composition and glucose metabolism in children.

Recently, a systematic review and meta-analysis on the association of FFM with IR or metabolic syndrome in children has been conducted (31). The study identified lower values of FFM/lean body mass (%) in children and adolescents with IR/glucose tolerance/metabolic syndrome and higher values of FFM/lean body mass when these are expressed in kg. Another recent study on the relationship between pediatric obesity and its cardiometabolic consequences has been explored (32). The study showed that the rates of impaired glucose tolerance and low insulin sensitivity increased progressively across quintiles of uric acid in youth with overweight/obesity, and impaired fasting glucose and IR were associated only with the highest quintile of uric acid. Takase et al. reported that FFM was related to HbA1c levels when the study excluded participants who had been identified as having have diabetes (33). Ghachem et al. showed that independently of age, FFM was an independent predictor of IR (34). However, more research on the association of FFM with glucose metabolism in children is still needed.

Several limitations need to be addressed. First, due to limited conditions, we only searched MEDLINE/PubMed, EMBASE, SinoMed, and the Cochrane Library, and only those studies published in English or Chinese were involved. There was a paucity of studies on FFM and glucose metabolism in children and adolescents, and there might have therefore been some omissions. Second, heterogeneity among studies was significant, which may be partially explained by the variety in assays used for the measurement of FFM in the studies. Third, there was a possibility of publication bias among the included trials. Fourth, because of the relatively small number of studies, we could not execute a stratified analysis of the relationship between FFM and glucose metabolism in children and adolescents. We noted a transient elevation in plasma glucose in adolescence, and studies have revealed that in normal healthy children FFM increased with age in boys, particularly during puberty. Adolescence—particularly in the early and middle periods—was also reported to be associated with insulin resistance directed toward carbohydrate metabolism, and with higher insulin levels than those found in younger children. This may be due to higher levels of growth hormone or other hormones that include pubertal hormones, or it may relate to an aspect of body mass. Unfortunately, puberty was not a weighted factor in the present analysis because of our limited access to raw data. Fifth, because FFM with age differs between men and women, it is necessary to consider the relationship between FFM and glucose metabolism in different age groups. Sixth, although BIA possesses several limitations, we included BIA-based studies in our meta-analysis because of the limiting conditions. This is a potential limitation of the study.

In the future, prospective cohorts with standardized assay measurements are needed to ascertain the causality between FFM and glucose metabolism in children and adolescents.

## Conclusion

From this systematic review and meta-analysis, we found that there is a positive relationship between FFM and fasting plasma insulin levels. This is the first known systematic review and meta-analysis to determine the associations of FFM with glucose metabolism in children and adolescents. Our results provided novel information with which to fill a gap in the literature in this area. Further examination of these relationships requires the execution of additional cohort studies.

## Data Availability Statement

The original contributions presented in the study are included in the article/supplementary material, further inquiries can be directed to the corresponding author/s.

## Author Contributions

FC conceived and designed the study. FC, JL, and DH conducted the systematic search, screened articles, and selected eligible articles. LW and FC wrote the initial draft of the manuscript, with revisions by all authors. FC, TL, YC, and ZL reviewed the manuscript and performed the analyses. All authors contributed to the article and approved the submitted version.

## Funding

This work was supported by the National Key Research and Development Program of China (2016YFC1300100), Public service development and reform pilot project of Beijing Medical Research Institute (BMR2019-11), and Beijing Municipal Administration of Hospitals Incubating Program (Px2022052).

## Conflict of Interest

The authors declare that the research was conducted in the absence of any commercial or financial relationships that could be construed as a potential conflict of interest.

## Publisher's Note

All claims expressed in this article are solely those of the authors and do not necessarily represent those of their affiliated organizations, or those of the publisher, the editors and the reviewers. Any product that may be evaluated in this article, or claim that may be made by its manufacturer, is not guaranteed or endorsed by the publisher.
